# Field evaluation of alphacypermethrin in indoor residual spraying for leishmaniasis control in an endemic area, northern Morocco

**DOI:** 10.1186/1756-3305-6-354

**Published:** 2013-12-13

**Authors:** Chafika Faraj, El Bachir Adlaoui, Souad Ouahabi, Mohammed Elkohli, Mohammed Elrhazi, Lhousseine Laqraa, Btissam Ameur

**Affiliations:** 1 Laboratoire d’Entomologie Médicale, Institut National d’Hygiène, 27 Avenue Ibn Batouta, Agdal, Rabat 10090, Morocco; 2 Service de lutte Antivectorielle, Direction de l’Epidémiologie et de Lutte contre les Maladies, Agdal, Rabat 10080, Morocco

**Keywords:** Cutaneous lehmaniasis, Control, IRS, Alphacypermethrin, Sand flies, Morocco

## Abstract

**Background:**

In Morocco, the main strategies of leishmaniasis vector control are based on environmental modifications. Use of local residual indoor spraying with synthetic pyrethroids is often envisaged. The need to evaluate this control method is essential. The current study was conducted to determine the efficacy of an alphacypermethrin IRS program against leishmaniasis vectors in an endemic area in the north of Morocco.

**Methods:**

The survey was conducted in four neighbouring localities in three different districts in northern Morocco: Ait Chaib and Aichoun in Sefrou district, Bouassem (Boulmane) and Lmrouj (Taounate). Indoor residual spraying with alphacypermethrin at a dose of 30 mg/m^2^ was used in Ait Chaib and Lmrouj localities during 2010, 2011 and 2012, while localities of Aichoun and Bouassem were taken as control. In the four studied areas, sand flies were collected bimonthly from April to November in 2011 and 2012, using sticky traps, to determine their abundance and feeding pattern. Alphacypermethrin IRS were evaluated for their residual effect using the WHO cone bioassay test. Leishmaniasis incidence was estimated by passive and active case detection in each study area.

**Results:**

Significant reductions in leishmaniasis incidence and in gravidity rate were observed when comparing sprayed and unsprayed localities. The residual activity of alphacypermethrin at the concentration used lasted 10 weeks after spraying. However, the abundance of sand flies was not significantly affected by alphacypermethrin IRS.

**Conclusion:**

This study indicated that IRS has a significant impact on leishmaniasis transmission; therefore it could be recommended as an effective tool for leishmaniasis control in areas with high leishmaniasis transmission.

## Background

Cutaneous Leishmaniases are endemic in Morocco with two described forms; anthroponotic and zoonotic leishmaniasis.The zoonotic cutaneous leishmaniasis caused by *Leishmania major* is endemic especially in the south of the country where its vector, *Phlebotomus papatasi* and its reservoir host, *Meriones shawi*, are prevalent [1]. The anthroponotic cutaneous leishmaniasis, due to *L. tropica* and transmitted by *Ph. sergenti*[2], is endemic in the center and the north of the country [3]. These infections represent a serious health problem in Morocco and have reached epidemic levels, in recent years, in many regions [4]. In 2011, the Moroccan Ministry of Health (MMOH) reported 4319 cutaneous leishmaniasis cases caused by both *L. major* and *L. tropica*[5].

Control measures against leishmaniasis rely both on case management, rodent reservoir control in zoonotic foci, and vector control in anthroponotic foci. Vector control methods include environmental modifications, use of bednets and local residual indoor spraying with synthetic pyrethroids. However, the impact of these measures was never investigated under field conditions within the biogeographical area treated.

The aim of this study is to evaluate entomological and epidemiological impacts of a leishmaniasis control program, conducted by MMOH, using a pyrethroid in indoor residual spraying (IRS) in an endemic area in the north of Morocco.

## Methods

### Study area

**Figure 1:**
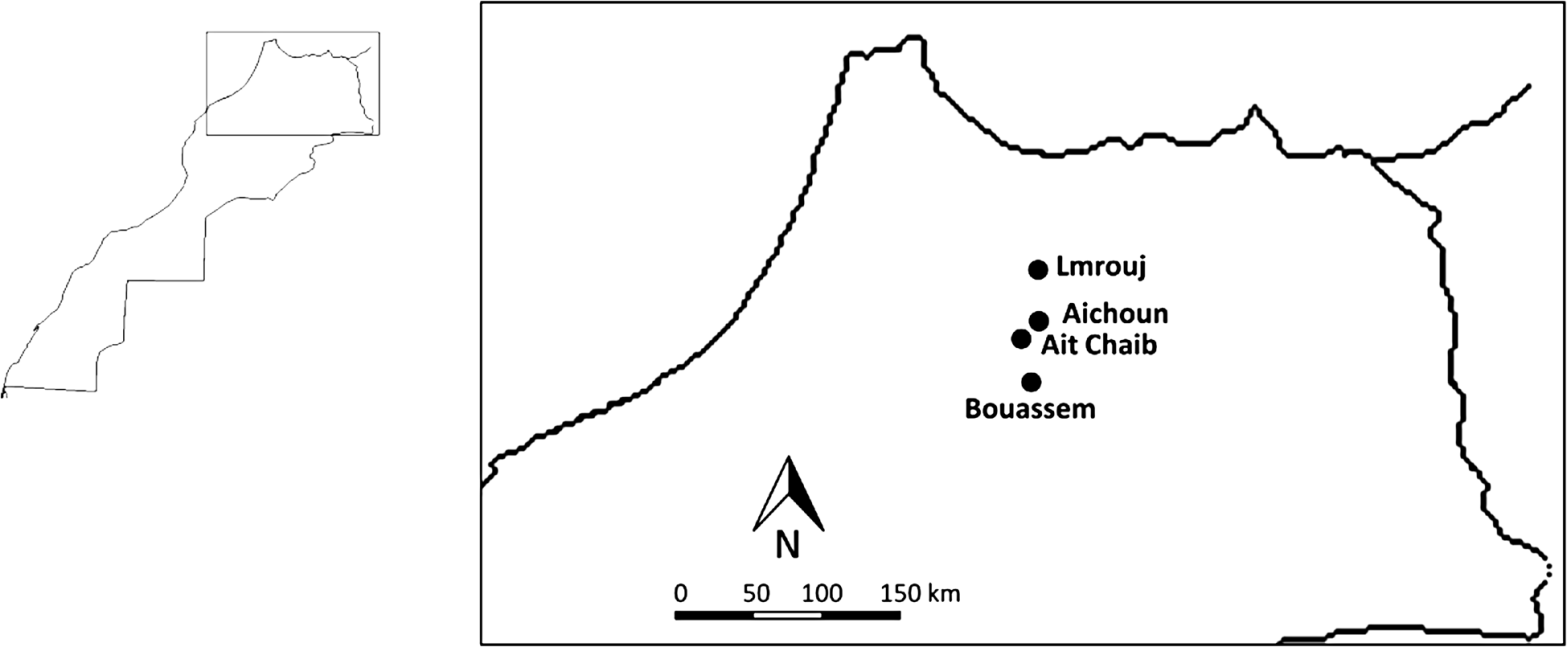
Map showing the study sites.

The survey was conducted in four neighbouring localities located in three different districts in northern Morocco: Ait Chaib and Aichoun in Sefrou district, Bouassem (Boulmane) and Lmrouj (Taounate) (Figure 1). The localities were chosen because they had similar topographical and epidemiological characteristics. The region is mountainous, with altitudes ranging from 900 to 1100 m. These localities have essentially a semi arid climate. There is rain from November to April. The annual mean rainfall is about 500 mm, annual mean temperature is 16°C with a mean maxima of 25°C and a mean minima of 9.5°C. August is usually the hottest month of the year.

The inhabitants of the four localities are, mainly farmers and breeders. Bovines, ovines, caprines, equines, dogs and chickens are common animals. The houses in all areas are generally built in a similar shape. Most of them are very simple and mainly made of bricks. Shelters for domestic animals are generally around the houses.

### Epidemiological data

Leishmaniasis incidence was measured by passive and active case detection in each study area. In addition to passive case detection made systematically by MMOH services, campaigns for active case detection were conducted in schools to identify further cases who may not be presenting at health facilities. These campaigns were carried out during March, September and December 2010, 2011 and 2012 in each study area.

Taking into account the seasonality and the transmission period in Morocco, we used an epidemiological year (July to June) for epidemiological data.

### Insecticide spraying

Indoor residual spraying with alphacypermethrin was conducted in Ait Chaib including 184 houses and Lmrouj (187 houses) while localities of Aichoun (225 houses) and Bouassem (576 houses) were taken as control.

On the basis of previous works, delimitating transmission period from May to October, IRS operations were planned for May of each year, but because of operational harms, they took place only in the end of June 2010, 2011 and 2012 in Ait chaib locality and in the beginning of July 2010, 2011 and 2012 in Lmrouj locality. Used insecticide is alphacypermethrin (10% SC) at a dose of 30 mg/m^2^. Each round, over 90% of households in both localities were treated. All indoor surfaces, including roof structures, animal shelters and caves near of houses were sprayed. The IRS operations were carried out by volunteer workers recruited from the local community and trained by the MMOH team. The treatment of the walls was done with a manual pressure sprayer of GLORIA type. Before treatment, workers were protected with a long sleeved overall, goggles, mask, gloves, boots and a helmet for safety.

### Entomological data

The entomological evaluation was based on the follow-up of the abundance of flies and their feeding rate in the sprayed and unsprayed villages.

Insecticide effectivness was evaluated by the follow-up of the insecticidal residual effect on the treated surfaces after assessing *Ph. sergenti* susceptibility to pyrethroids.

#### Sand fly sampling

Systematic sand fly collections, using the sticky trap method, were carried out bimonthly inside animal shelters from April to November 2011 and during the same period of 2012. Five stations were chosen at random in each locality and 10 traps were placed in each station.

All sand flies were sorted and assigned to species based on morphological characters using standard identification keys [6]. The female sand flies were classified according to the state of their abdomens (unfed, fed or gravid).

#### Insecticide persistence

The efficacy and the residual action of the insecticide against the local sand fly vectors under field conditions were assessed using WHO recommended procedures [7]. Contact bioassy cones were set against sprayed walls in Ait chaib locality, the day after spraying then once every two weeks until the loss of efficiency (when mortality rate of sand flies exposed to sprayed walls became inferior to 70%). Bioassay was carried out with field sand flies caught in the Aichoun locality. Sand flies were exposed to alphacypermethrin treated surfaces in the transparent plastic bioassay cone for 30 min. In each assay, 12 cones were fastened on walls at 1.5 m from the ground in 4 treated households (with 3 cones per house). 4 cone chambers were used as control and processed in the same way, by exposing sand flies to untreated walls. In each cone, test batches of fifteen sand flies were introduced by an aspirator. After exposure, sand flies were removed with a bent-end aspirator, through the opening of the chamber, and immediately transferred to holding pots closed with a fine mesh and provided with glucose solution for nourishment. They were kept at 20–25°C to score the mortality rates after 24 hours.

#### Sand fly susceptibility to pyrethroids

Resistance to pyrethroids was assessed on sand fly populations from Aichoun locality (control locality) by using standard WHO testing procedures [8].

Sand flies were collected using CDC Light Traps in animal’s shelters on June 2012. As we did not have alphacypermethrin impregnated papers available, four lots of about 20 sand flies were exposed to another pyrethroid (lambdacyhalothrin 0.05%). A control test was performed, simultaneously, using control papers. Knockdown rates were noted at 5 min intervals during the insecticide exposure. After one hour of exposure, sand flies were transferred to the observation tube and kept in appropriate conditions (25 ± 2°C and 80% ± 10% relative humidity) for 24 hours. Sufficient relative humidity was ensured by putting small pieces of cotton wool impregnated with distilled water on the top of the cups. After this period, mortality rates were calculated.

#### Data analysis

Data were analyzed using Genstat (15^th^ edition). Pearson chi-square (χ2) test was applied to compare incidence rates before and after intervention and between treated and control localities. The same test was also used to compare the gravidity rate across study areas. The significance level was set at 5%.

Reduction in sand fly abundance due to spraying was estimated by the formula of Mulla *et al.*[9]:

$$\%\;\mathrm{of}\;\mathrm{reduction}=100-\left(\left(C1/T1\right)\times \left(T2/C2\right)\times 100\right)$$

C1: sand fly abundance before spraying date in control localities

C2: sand fly abundance after spraying date in control localities

T1: sand fly abundance before spraying in treated localities

T2: sand fly abundance after spraying in treated localities

C1 and T1 have been estimated considering the mean of sand fly abundance over the month before spraying date.

C2 and T2 correspond to the mean of sand fly abundance over the 10 weeks following the spraying date (this period corresponds to the duration of the residual effect of alphacypermethrin in this study).

## Results

### Epidemiological impact of intervention

Table 1 shows the evolution of leishmaniasis incidence in treated and control localities.

**Table 1:** Cutaneous leishmaniasis incidence (/1000) in treated and control localities

** Arm**	**Localities**	**2010-2011**	**2011-2012**	**2012-2013**
Treated	Mrouj	10.32	1.29	0.00
	Ait chaib	2.92	00	0.00
	Total	6.85	0.68	0.00
		[2.63-59.02]	[0.41-8.84]	
Control	Aichoun	8.98	3.37	5.61
	Bouassem	13.57	2.86	10.71
	Total	11.79	3.06	8.72
		[1.45-40.44]	[0.16-6.36]	[1.61-40.56]

Between [ ]: 95% confidence intervals.

Before spraying there was an incidence of 7 per 1000 cases in sprayed localities; this dropped significantly (p < 0.001) to 0 per 1000 after two spraying cycles. After IRS, CL incidence in the intervention area was significantly (p < 0.001) lower than in unsprayed localities (0 vs 9 per 1000).

### Entomological impact of intervention

#### Sand fly abundance

During two years of capture (2011–2012), 3315 sand flies were collected (in the four localities) and identified. They comprised seven species: *Ph. sergenti*, *Ph. longicuspis*, *Ph. perniciousis*, *Ph. papatasi*, *Ph. ariasi*, *Sergentomyia minuta* and *S. fallax. Ph. sergenti* was the most prevalent species in the four areas, it constitutes 87.0%, 50.4%, 73.4%, and 64.9% of total sand flies collected in Aichoun, Bouassem, Ait Chaib and Mrouj respectively (Table 2).

**Table 2:** Number of sand flies collected in the four localities during the two study years

	**Aichoune**			**Bouassem**			**Ait Chaib**			**Mrouj**			**Total**
	**Male**	**Female**	**%**	**Male**	**Female**	**%**	**Male**	**Female**	**%**	**Male**	**Female**	**%**	
*Ph. sergenti*	1501	256	87.0	261	76	50.4	155	22	73.4	192	62	65.8	2525
*Ph. longicuspis*	36	8	2.2	45	4	7.3	18	2	8.3	82	41	31.9	236
*Ph. perniciosus*	88	17	5.2	219	51	40.4	37	2	16.2	5	3	2.1	422
*Ph. papatasi*	85	6	4.5	3	0	0.4	4	1	2.1	0	0	0.0	99
*Ph. ariasi*	0	0	0.0	3	0	0.4	0	0	0.0	1	0	0.3	4
*S. minuta*	2	0	0.1	3	0	0.4	0	0	0.0	0	0	0.0	5
*S. fallax*	7	13	1.0	2	2	0.6	0	0	0.0	0	0	0.0	24
*Total*	1719	300	100	536	133	100	214	27	100	280	106	100	3315

**Figure 2:**
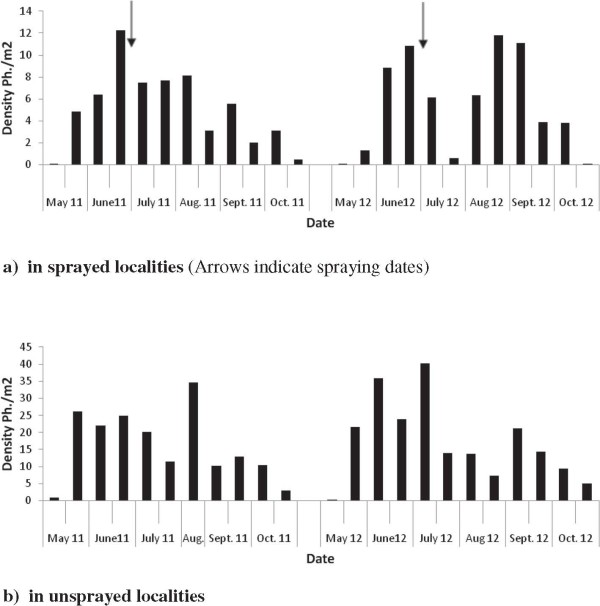
**Sand fly abundance evolution during 2011–2012. a)** in sprayed localities (Arrows indicate spraying dates). **b)** in unsprayed localities.

Figure 2 shows the evolution of mean sand fly abundance in each study arm. Generaly, sand fly activity starts from May and lasts to October. Abundance of sand flies, both in control and treated localities presented two well marked peaks. The first occurred in May-June and the second in August-September.

Overall, there was no differences in the evolution of sand fly densities in control and treated localities. Compared to control arm, sprayed localities showed a slight reduction in sand fly abundance during 2011 (8%) while an increase in abundance (79%) was observed during 2012 (Table 3).

**Table 3:** Reduction rate of sand fly abundance in treated localities in comparison to that of control localities

**Year**	**C1**	**C2**	**T1**	**T2**	**Abundance reduction(%)**
2011	26.1	19.6	9.3	6.4	08%
2012	27.9	14.1	6.6	5.9	−79%

### Sand fly gravidity rate

Before intervention (May-June), three females from a total of 40 (7.5%) were gravid in the IRS arm (8% in Ait chaib and 6% in Mrouj). After IRS, gravidity was significantly reduced from 7.5 to 0% (p = 0.008) (Table 4).

**Table 4:** Gravidity rate of female sand flies observed before and after IRS in treated and control areas

		**Before spraying**		**After spraying**	
		**Dissected (gravid) females**	**Gravidity% [95% CI]**	**Dissected (gravid) females**	**Gravidity% [95% CI]**
Control	Aichoun	44 (3)	7	95 (11)	12
	Bouassem	67 (7)	10	34 (5)	15
	Total	111 (10)	9.1	129 (16)	12
			[0.7-59.4]		[3.3-36.9]
Treated	Mrouj	16 (1)	6	70 (0)	0
	Ait chaib	24 (2)	8	17 (0)	0
	Total	40 (3)	7.5	87 (0)	0
			[1.3-33.6]		

In the control arm, the gravidity rate increased from 9% in May-June to 12% in July-August.

Difference in gravidity rates between the overall control and treated areas after spraying (12% vs 0%) is significant (p < 0.001).

### Insecticide residual activity

Results of bioefficacy test showed satisfactory percentages of mortality one day after spraying (89.3%); but this decreased progressively with weeks: 84.4%, 82.9%; 80.5, 78.2 and 72.8% after 2, 4, 6, 8 and 10 weeks after intervention, respectively (Table 5). The residual activity of the insecticide three months after the spraying was only 61.45%.

**Table 5:** **Mortality rates of *Phlebotomus sergenti *** **females in the bioassay cones in Ait Chaib locality**

**Time**	**Exposed sand flies (dead)**	**Mortality (%)**
Pre spraying	171 (11)	06.43
Post spraying		
01 day	178 (159)	89.32
02 weeks	180 (152)	84.44
04 weeks	175 (145)	82.86
06 weeks	170 (137	80.59
08 weeks	179 (140)	78.21
10 weeks	180 (131)	72.78
12 weeks	179 (110)	61.45

#### *Ph sergenti* susceptibility to pyrethroids

Results of wild collected *Ph. sergenti* susceptibility tests to lambdacyhalothrin are presented in Table 6.

**Table 6:** **Insecticide susceptibility tests of *Phlebotomus sergenti *** **to lambdacyhalothrin**

**Exposed sand flies**	**Mortality (%)**	**KDT50 (min)**	**KDT90 (min)**
76	100	11	20

Lambdacyhalothrin induced a *Ph. sergenti* knockdown of 100% after less than 30 min of contact and no specimen survived after 60 min exposure. Tested populations of *Ph. sergenti* showed a normal susceptibility to this insecticide.

## Discussion

The results presented here show that in areas with low leishmaniasis incidence (<15 per 1000), indoor residual spraying with alphacypermethrin can lead to a significant reduction in leishmaniasis transmission. In both localities under intervention, leishmaniasis incidence and gravidity rate have been significantly reduced. However, sand fly abundance was not significantly affected. In fact, sand flies were collected from animal shelters, which are also reproduction, resting and blood feeding sites. As IRS is directed exclusively against adult stages, larvae will not be affected until the emergence of adults and their contact with treated surfaces. Once on the walls, young blood fed adults are killed and do not have time to rest inside to digest their blood meal. It is known that sand fly larval development is slow and uneven mainly under temperate conditions [10,11]. The emergence of young flies that belonged to the same batch of eggs may extend over a long period (up to two months). The continuous emergence of adults continually replenished the population.

Similar results were observed in Brazil by Passerat de Silans *et al*. [12] who showed that residual insecticide applications of cypermethrin had no effect on the overall density of peridomiciliar *Lutzomyia longipalpis* populations. Alexander *et al*. [13] also found that residual spraying of walls with delthamethrin in a Colombian village surrounded by forest had no perceptible effect on the number of sand flies entering houses; although the insecticidal activity of the treated surfaces was undiminished during the study period. While other authors reported that residual pyrethroid spraying reduces sand fly indoor density in various localities in South America [14-16] and China [17] probably, because of their repulsive effect.

The residual effects of pyrethroid insecticides reported in the literature are variable according to formulations, doses used, types of surfaces tested and species of sand flies exposed. In this program, the residual effect of alphacypermethrin, at a dose of 30 mg/m^2^, remained for less than three months (about 10 weeks). A shorter duration was reported by Morsy *et al*. [18] in Egypt where the exposition of *Ph. papatasi*, during 30 min, on cement walls treated with permethrin, 75 days after treatment involved only 51.9% of mortality. A similar duration was reported by Passerat De Silans *et al*. [12] who indicated that residual activity of cypermethrin against *Lu. longipalpis* in Brazil was limited to two months.

Longer periods were also reported in rural areas of the new World tropics. Le Pont *et al*. [14] reported that one deltamethrin spray application in Bolivia was enough to eliminate *Lu. longipalpis* from the dwelling during 9 months. Falcao *et al*. [15] demonstrated that the residual action of deltamethrin remained for more than one year on walls of concrete and lime painted in a Brazilian focus of American cutaneous leishmaniasis. Davies *et al*. [16] confirmed that, with a concentration of 25 mg/m^2^ of lambdacyhalothrin, the insecticide remained 100% lethal for the sand fly *Lu verrucarum* for up to 6 months in endemic villages of Peru.

Hence, because of the low residual activity of alphacypermethrin (less than three months) and the long sand flies development period in Morocco (more than 6 months), one spray round per year is not sufficiently powerful to control sand fly populations in many regions of Morocco. Indeed, sand fly populations peaked twice during the season, at the end of spring: May-June and again at the end of summer: August-September [19]. This bi-modal population density pattern is characteristic of *Ph. sergenti* in semi arid habitats and has to be taken into account when determining the best application schedule of insecticide. Ambient conditions (temperature and humidity) were more favourable to transmission during periods corresponding to the second peak. Insecticide persistence should largely cover this period.

Application of insecticide during the two years of the study in the two localities has taken place after the first peak (end of June). Insecticide remained effective during the following months (July-August). The insecticide has no more efficacy at the beginning of September, when conditions become favourable to transmission. This suggests that two spray rounds are needed to maintain good effectiveness. The first should begin early in the season before the first peak of sand fly populations early in May (in order to kill the maximum number of sand flies throughout the whole season) and the second round early in August. However, such a strategy implicates an increase in the cost of leishmaniasis prevention and cannot be implemented everywhere. It must be reserved only for areas with the highest leishmaniasis transmission.

## Conclusion

This study indicated that IRS has a significant impact on leishmaniasis transmission; therefore it could be recommended as an effective tool for leishmaniasis control in areas with high leishmaniasis transmission.

## Competing interests

The authors declare that they have no competing interests.

## Authors’ contributions

CF conceived and designed the study and drafted the manuscript. EA carried out the data analysis and participated in the review of the manuscript. SO Carried out sand fly identification and participated in the review of the manuscript, ME, LL, ME carried out the field work and the bioassay tests. BA participated in study design and in the review of the manuscript. All authors read and approved the final version of the manuscript.

## References

[B1] Rioux JA Trente ans de coopération franco-marocaine sur les leishmanioses: Dépistage et analyse des foyers. Facteurs de risque. Changements climatiques et dynamique nosogéographique Assoc des Anciens Elèves de l’Institut Pasteur 2001 6 90 101

[B2] Guilvard E Rioux JA Gallego M Pratlong F Mahjour J Martinez-Ortega E Dereure J Saddiki A Martini A *Leishmania tropica* au Maroc III-Rôle de *Phlebotomus sergenti*. A propos de 89 isolats Ann Parasitol Hum Comp 1991 6 96 99 1776784 10.1051/parasite/199166396

[B3] Rhajaoui M Les leishmanioses humaines au Maroc: une diversité nosogéographique Pathol Biol 2011 6 4 226 229 http://www.sciencedirect.com/science/article/pii/S0369811409001813 10.1016/j.patbio.2009.09.003 19942371

[B4] Faraj C Ouahabi S Adlaoui E Elkohli M Lakraa L Elrhazi M Ameur B Insecticide susceptibility status of *Phlebotomus* (*Paraphlebotomus*) *sergenti* and *Phlebotomus* (*Phlebotomus*) *papatasi* in endemic foci of cutaneous leishmaniasis in Morocco Parasit Vectors 2012 6 51 doi:10.1186/1756-3305-5-51. http://www.parasitesandvectors.com/content/pdf/1756-3305-5-51.pdf 10.1186/1756-3305-5-51 22429776 PMC3359231

[B5] Moroccan Ministry of Public Health Etat d’avancement des Programmes de Lutte contre les Maladies Parasitaires 2012 Rabat: Direction de l’Epidémiologie et de Lutte contre les Maladies

[B6] Lewis DJ Phlebotomine sand flies (Diptera. Psychodidae) of the Oriental Region Bull Br Mus Nat Hist Entomol Ser 1978 6 217 343

[B7] World Health Organization Insecticide resistance and vector control Seventeeth Report of the WHO Expert Committee on Insecticides, World Health Organ Tech Rep Ser 443 1970 Geneva: WHO 4986494

[B8] World Health Organization Instructions for determining the susceptibility or resistance of adult blackflies, sand flies and biting midges to insecticides 1981 Geneva: WHO/VBC/81.810, WHO

[B9] Mulla MS Norland RL Fanara DM Darwazeh HA McKean DW Control of chironomid midges in recreational lakes J Econ Entomol 1971 6 300 307

[B10] Schmidt ML Laboratory culture of two Phlebotomus species, *P. papatasi* and *P. orientalis* Bull World Health Organ 1964 6 577 578 14272472 PMC2555045

[B11] Killick-Kendrick R Phlebotomine vectors of the leishmaniases: a review Med Vet Entomol 1990 6 1 24 10.1111/j.1365-2915.1990.tb00255.x 2132963

[B12] de Passerat Silans LNM Dedet JP Arias JR Field monitoring of cypermethrin residual effect on the mortality rates of the phlebotomine sand fly *Lutzomyia longipalpis* in the state of Paraiba Brazil.Mem Inst Oswaldo Cruz 1998 6 339 344 10.1590/S0074-02761998000300012 9698867

[B13] Alexander B Jaramillo C Usma MC Roa W Travi BL An Attempt to control phlebotomine sand flies (Diptera: Psychodidae) by residual spraying with delthamethrin in a Colombian village Mem Inst Oswaldo Cruz 1995 6 421 424 10.1590/S0074-02761995000300020 8544745

[B14] Le Pont F Padilla JM Desjeux P Richard A Mouchet J Impact de pulverisations de deltamethrine dans un foyer de leishmaniose de Bolivie Ann Soc Belg Med Trop 1989 6 223 232 2610530

[B15] Falcao AL Falcao AR Pinto CT Gontijo CM Falqueto A Effectof deltamethrin spraying on the sand fly populations in a focusof American cutaneous leishmaniasis Mem Inst Oswaldo Cruz 1991 6 399 404 10.1590/S0074-02761991000400004 1842430

[B16] Davies CR Llanos-Cuentas EA Campos P Monge J Leon E Canales J Spraying houses in the Peruvian Andes with lambdacyhalothrin protects residents against cutaneous leishmaniasis T Roy Soc Trop Med H 2000 6 631 636 10.1016/S0035-9203(00)90214-1 11198646

[B17] Xiong G Jin C Studies on deltamethrin in the control of peridomestic wild *phlebotomus chinensis* Chinese Journal of Parasitology and Parasitic Diseases 1987 6 176 179 3447777

[B18] Morsy TA Aboul-Ela RG El-Gozamy BMR Salama MMI Ragheb DA Residual effects of four insecticides applied for indoor control of *Phlebotomus papatasi* (Scopoli) J Egypt Soc Parasitol 1993 6 485 492 7690827

[B19] Faraj C Adlaoui E Ouahabi S El Kohli M El Rhazi M Lakraa L Ameur B Distribution and bionomics of sand flies in five ecologically different cutaneous leishmaniasis foci in Morocco ISRN Epidemiology 2013 8 http://www.hindawi.com/isrn/epidemiology/2013/145031/

